# Enlarging aneurysm sac post EVAR – type V or occult type II Endoleak?

**DOI:** 10.1186/s42155-023-00348-z

**Published:** 2023-02-07

**Authors:** Shyamal Patel, Joo-Young Chun, Robert Morgan

**Affiliations:** St George’s Hospital NHS Foundation Trust, Blackshaw Road, Tooting London, UK

## Abstract

**Purpose:**

Several theories exist regarding the underlying mechanism of type V endoleaks (T5EL), which remains unclear. Torikai et al. (2018) describe sac expansion in cases with patchy heterogenous enhancement of peripheral thrombus and postulate these are due to atypical type II endoleaks (T2EL) from proliferated vasa vasora. These cases of apparent endotension pose a therapeutic challenge as continued sac expansion warrants active intervention.

**Materials and methods:**

Retrospective review of T5EL cases was performed who underwent multidisciplinary discussion at our institution between 2020–2021. Clinical history and imaging were reviewed by a vascular interventional radiologist aiming to identify the underlying mechanism of sac expansion.

**Results:**

Two cases of these specific T5ELs were identified. One patient underwent endovascular management and image-guided aspiration of intra-sac fluid whilst another underwent open surgical ligation and sac plication. In both cases, fluid re-accumulated with re-expansion of the aneurysmal sac on follow-up. Careful review of CT imaging showed subtle foci of peripheral sac enhancement, suggestive of vasa vasora causing occult T2ELs. This was not visible on single phase CTA, super-selective angiography or cone beam CT.

**Conclusion:**

We identified two complex cases with unexplained sac expansion following EVAR suggestive of T2ELs from proliferated vasa vasora. Transcatheter embolisation of this network of vessels although challenging has been previously considered to stunt sac expansion. We suggest this phenomenon is under-diagnosed. Nevertheless, long-term surveillance is warranted as continued sac expansion risks changes in aneurysm morphology leading to potential loss of the proximal/distal seal zones.

## Introduction

Type V endoleaks (T5ELs) describe aneurysm sac expansion after endovascular aortic repair (EVAR) without a demonstrable type I-IV endoleak (Chaikof et al. 2018; Gilling-Smith et al. 1999; White and May 2000). Originally T5ELs occurred with early iterations of the Gore Excluder aortic grafts (Gore, Flagstaff, AZ) proposedly from porosity of the graft fabric, leading to its modification in the early noughties.

However, persistence of T5ELs with newer generation endografts has led to several hypotheses including intermittent or low-flow endoleaks not visualised on imaging (Sambeek et al. 2004; Torres-Blanco and Miralles-Hernández 2021; Parsa et al. 2021). Other authors postulate hyperfibrinolysis and local coagulation activation as the mechanism of a clear gelatinous material accumulating within the aneurysm sac, known as sac hygroma (Risberg et al. 2001, 2004). Torikai et al. (2018) propose proliferation of vasa vasorum in the aortic wall causes occult type II endoleaks (T2EL) suggested by patchy heterogenous enhancement of sac thrombus on delayed-phase CT (Torikai et al. 2018). These cases of apparent endotension pose a challenge as continued sac expansion warrants active intervention (Risberg et al. 2004; Deery et al. 2018).

We present two cases of patients with enlarging aneurysm sacs but no definable endoleak on imaging, which we propose are type II endoleaks secondary to reversal of flow in the vasa vasora.

## Materials and methods

Cases of T5EL with heterogenous appearances of the aneurysm sac that underwent multidisciplinary discussion at our tertiary referral centre between 1^st^ January 2020 and 31^st^ December 2021 were identified. Retrospective data analysis of all electronic patient records (EPR)/imaging was performed and reviewed by a vascular interventional radiologist in attempts to identify a potential mechanism for sac expansion. For this type of study formal ethical approval is not required.

## Results

Two cases of the specific subtype of T5EL were identified in male patients aged 84–85 years. Both patients underwent elective EVAR with Zenith endografts (Cook Medical, Bloomington, USA) between 2007–2010 for infra-renal abdominal aortic aneurysms and later presented with persistent aneurysm sac expansion. No endoleak was identified on CTA nor were there any inflammatory changes around the aneurysm sac. Both patients underwent diagnostic catheter angiography with cone beam CT, but a cause of sac expansion could not be identified. This included super-selective catheter angiograms scrutinising the SMA/internal iliac vessels for small T2ELs.

Both patients had been treated for other endoleaks in the past, prior to presentation with unexplained sac expansion. These were managed endovascularly by transcatheter embolisation of T2ELs or endograft relining with a Nellix endovascular aneurysm sealing system (Endologix, Irvine, USA) in the first patient for a type IIIb endoleak.

**Fig. 1:**
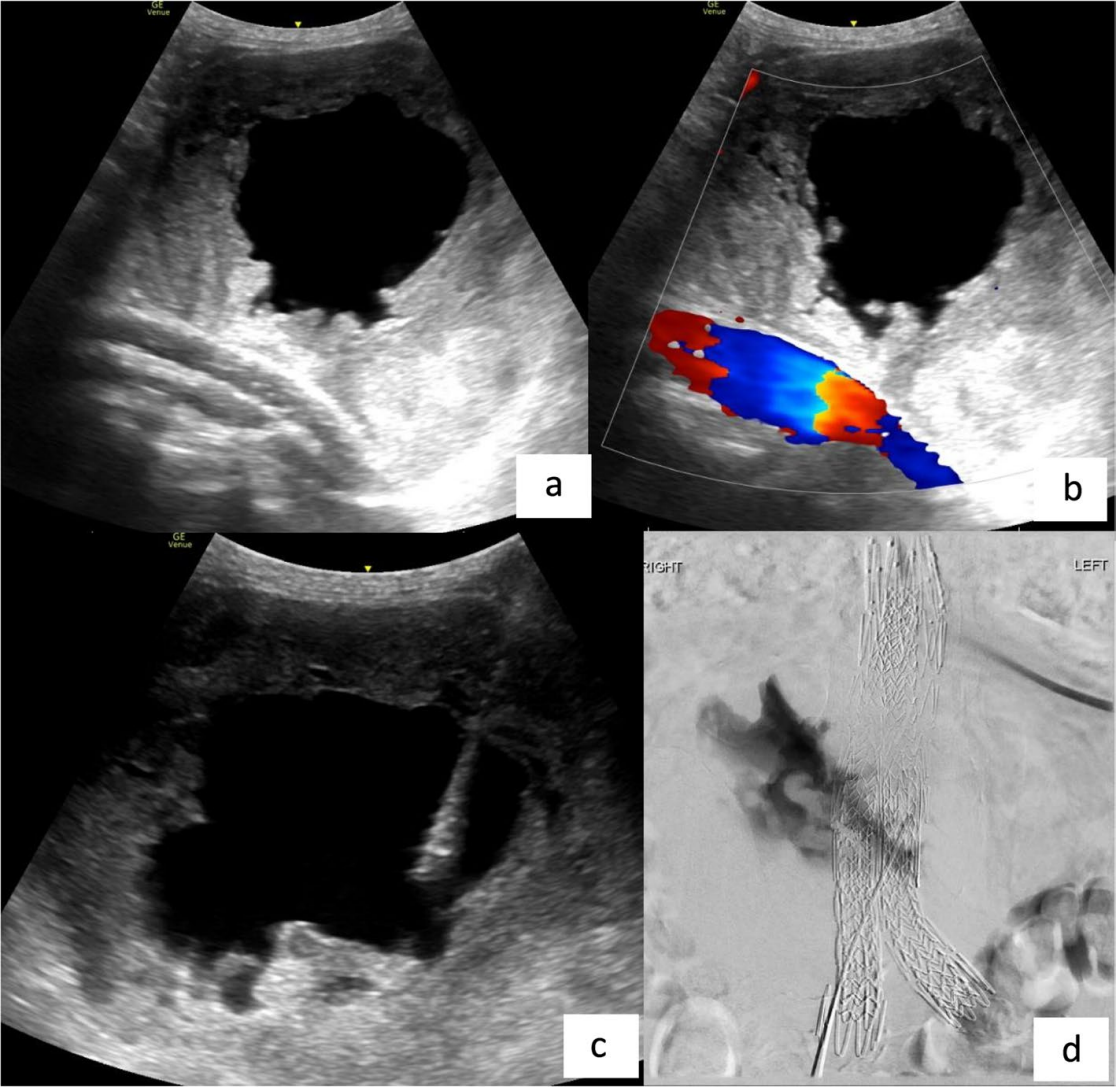
**a**—longitudinal view of the aneurysm sac with a central anechoic component surrounded by echogenic thrombus. The components of the Nellix endograft are seen deep to this. **b** – The endograft is patent with no endoleak identified. **c** – US guided direct puncture of central anechoic component of the aneurysm sac. **d** – Contrast injection into the aneurysm sac fills the fluid component without any retrograde flow into the aortic/iliac branches

**Fig. 2:**
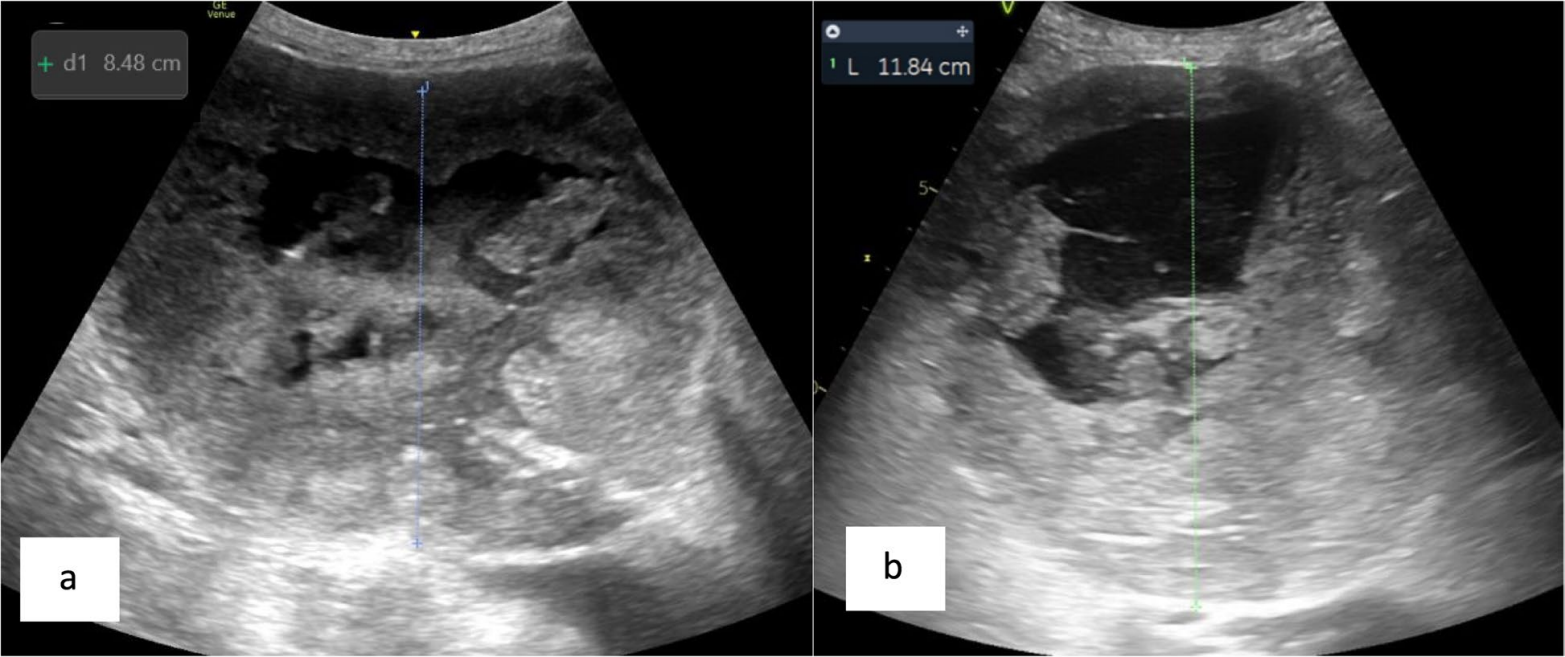
**a** Diameter and appearances of the aneurysm sac directly following aspiration. This can be compared with **b** (follow up study), where re-accumulation of the fluid component again results in dilatation of the aneurysmal sac

Duplex ultrasound (US) of the aneurysm sac in the first patient demonstrated a large central anechoic fluid component with peripheral echogenic thrombus and no endoleak (Fig. 1). Given these findings and due to persistent sac enlargement (12 cm in diameter from 9.5 cm at the time of last endovascular intervention), the patient underwent direct sac puncture with a 19G trocar needle. Serosanguinous fluid flowed out of the trocar, suggesting elevated sac pressure. Approximately 60mls of fluid was aspirated and an angiogram performed from within the sac did not opacify any aortic or iliac branches. The partly decompressed aneurysm sac measured 8.5 cm in diameter (Fig. 2a). Biochemical analysis of the fluid—albumin, protein and LDH levels were in the normal serum ranges. Cytology, microscopy and culture were also negative. Surveillance US 6 weeks post-procedure demonstrated re-accumulation of fluid centrally and re-expansion of the aneurysm sac to pre-procedure dimensions with no endoleak (Fig. 2b). The patient remains asymptomatic with stable aneurysm sac dimensions and therefore remains on active surveillance.

A T2EL endoleak from left lumbar arteries in the second patient was resistant to attempts at endovascular embolisation. Due to persistent sac expansion, the patient underwent open sac plication. Intraoperatively, T2ELs from previously embolised left lumbar arteries and the IMA were ligated. A small type IIIb endoleak from the main body was repaired with bioglue. Sac haematoma and formerly deployed embolic material was evacuated and the sac was plicated. Similarly, surveillance US found re-expansion of the sac to pre-operative dimensions without an endoleak. The sac size remained stable on surveillance. However, the patient presented a year later with aortic rupture due to disruption of the distal endograft seal zone and was treated by palliation with no further interventions.

**Fig. 3:**
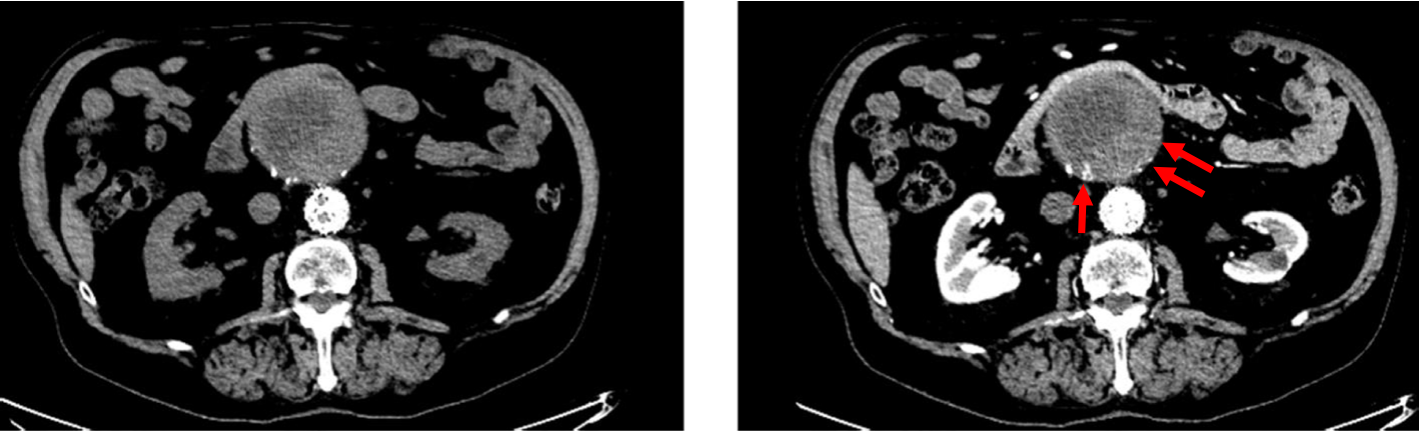
Pre and post contrast CT images of Patient 1 following embolisation of small T2EL which demonstrates enhancement of peripheral sac thrombus. Tiny foci of contrast opacification along the aortic wall (arrows) may represent vasa vasora

A careful review of pre and post contrast CTA in both patients showed subtle enhancement of sac thrombus adjacent to the aortic wall (Fig. 3), which is consistent with a T2EL due to retrograde flow from mural vasa vasora (Torikai et al. 2018). Interestingly, these areas of enhancement were absent on the CT performed when patient 2 presented with sac rupture, possibly due to loss of arterial pressure.

## Discussion

Patients with enlarging aneurysm sacs but no identifiable endoleaks are diagnosed as having T5ELs. These patients pose diagnostic and therapeutic challenges, as the underlying mechanism remains unclear. As T5ELs do not involve pressurised blood entering the aneurysmal sac, one could argue it carries a low rupture risk and active intervention should be reserved for symptomatic patients. The first patient we describe remains asymptomatic and under active surveillance. However, as with our second patient, continued expansion may alter the morphology of the aneurysm sac. This in turn risks disruption of the endograft seal zones and the development of secondary type I/III endoleaks.

We found that despite percutaneous aspiration and surgical plication, sac re-expansion occurred to pre-treatment dimensions. Other authors also report similar challenges. Derboghossian et al. (2020) (Derboghossian et al. 2020), described a similar patient with unexplained symptomatic sac expansion post-EVAR. Sonography found central anechoic fluid and peripheral echogenic thrombus with pressurised haemoserous fluid expelled from the sac during open exploration. Haematoma was evacuated and the sac was plicated. On 6-week follow-up US, the sac re-expanded with mostly central anechoic material. The authors speculated several hypotheses and considered ultrafiltration across semi-permeable graft material.

Williams (1998) reported a large cystic mass surrounding a PTFE prosthesis after open aneurysm repair. He found clear serous fluid with a protein content of serum and postulated this represented ultrafiltration across the graft (Williams 1998).

These cases may in fact represent atypical T2ELs involving retrograde flow from the vasa vasora. Insertion of covered stents has been shown to cause proliferation of vasa vasora from the adventitia to the intima (Sanada et al. 1998). Toriaki et al. (2018) postulated that this leads to neovascularisation of intrasaccular thrombus and an atypical T2EL from vasa vasora. Their main CT finding was subtle heterogenous contrast enhancement in the peripheral sac thrombus, which was only identified on delayed phase imaging (Sanada et al. 1998; Fikani et al. 2021).

On careful review of the images in our patients, subtle peripheral foci of arterial enhancement in the aneurysm wall (Fig. 3) could be appreciated on arterial phase images but only when the pre-contrast phase was available for comparison. CT protocols vary by institution with many not routinely performing pre-contrast or delayed phase imaging. This is comparable to practice in our department where post-EVAR imaging includes a CTA at 4–6 weeks. Further surveillance is at 3 and 6 months with US and at 12 months with a CTA. If there are no concerns, patients move to annual US thereafter. If there is increase in sac size/an endoleak identified on US, an urgent CTA is performed consisting of arterial phase images only with pre-contrast/delayed phases usually reserved for problem solving.

Furthermore, the vasa vasora are tiny vessels that do not opacify readily even on super-selective catheter angiography or cone-beam CT, as in our experience. Toriaki’s group used a combination of superselective angiography and on-table CT to confirm vasa vasorum involvement and perform successful embolisation. Unfortunately, such facilities are not available in all institutions.

Other reports of T5EL describe sac hygroma – a gelatinous material within the aneurysm sac (Risberg et al. 2001, 2004; Thoo et al. 2004). These are distinct from the serosanguinous fluid in our experience and Derboghossian et al. Risberg et al. (2001) described four such patients that underwent EVAR between 1995–2000 with earlier generation endografts (Gore excluder, Hemobahn, Zenith). Analysis of the gelatinous fluid showed hyperfibrinolysis and coagulation activation. As fabric from earlier endografts was more porous than later iterations, it is postulated these in fact represent type IV microleaks (Sambeek et al. 2004; Derboghossian et al. 2020). Interestingly, as with our findings, several treatment strategies including percutaneous aspiration, surgical fenestration or surgical resection of the sac were unsuccessful in preventing sac re-expansion.

## Conclusion

Our findings suggest the specific type of T5ELs we describe above actually represent occult T2EL from vasa vasora. We propose that this obscure phenomenon may go underdiagnosed, unsurprising given the diagnostic challenges we outlined above. In addition to unenhanced and delayed phase CTA, contrast-enhanced US of peripheral sac thrombus and super-selective angiograms of lumbar/inferior mesenteric arteries may be warranted to confirm or exclude this entity as a cause of unexplained sac expansion.

Long-term surveillance is essential to identify an occult source of endoleak and to maintain the original sealing zones. As for the optimal therapeutic intervention, this remains a challenge and a topic for discussion as experience accrues of this phenomenon.

## Data Availability

Available on request from the corresponding author.

## Abbreviations

EVAR: Endovascular aortic repair
T5EL: Type V endoleak
T2EL: Type 2 endoleak
